# Dynamic integration of enteric neural stem cells in ex vivo organotypic colon cultures

**DOI:** 10.1038/s41598-021-95434-4

**Published:** 2021-08-05

**Authors:** Georgina Navoly, Conor J. McCann

**Affiliations:** Stem Cells and Regenerative Medicine, UCL Great Ormond Street Institute of Child Health, 30 Guilford Street, London, WC1N UK

**Keywords:** Neural stem cells, Stem cells, Regeneration

## Abstract

Enteric neural stem cells (ENSC) have been identified as a possible treatment for enteric neuropathies*.* After in vivo transplantation, ENSC and their derivatives have been shown to engraft within colonic tissue, migrate and populate endogenous ganglia, and functionally integrate with the enteric nervous system. However, the mechanisms underlying the integration of donor ENSC, in recipient tissues, remain unclear. Therefore, we aimed to examine ENSC integration using an adapted ex vivo organotypic culture system. Donor ENSC were obtained from *Wnt1*^*cre/*+^*;R26R*^*YFP/YFP*^ mice allowing specific labelling, selection and fate-mapping of cells. YFP^+^ neurospheres were transplanted to C57BL6/J (6–8-week-old) colonic tissue and maintained in organotypic culture for up to 21 days. We analysed and quantified donor cell integration within recipient tissues at 7, 14 and 21 days, along with assessing the structural and molecular consequences of ENSC integration. We found that organotypically cultured tissues were well preserved up to 21-days in ex vivo culture, which allowed for assessment of donor cell integration after transplantation. Donor ENSC-derived cells integrated across the colonic wall in a dynamic fashion, across a three-week period. Following transplantation, donor cells displayed two integrative patterns; longitudinal migration and medial invasion which allowed donor cells to populate colonic tissue. Moreover, significant remodelling of the intestinal ECM and musculature occurred upon transplantation, to facilitate donor cell integration within endogenous enteric ganglia. These results provide critical evidence on the timescale and mechanisms, which regulate donor ENSC integration, within recipient gut tissue, which are important considerations in the future clinical translation of stem cell therapies for enteric disease.

## Introduction

Loss of neurons within the enteric nervous system (ENS) can impact nearly every region of the gastrointestinal tract, resulting in a wide variety of disorders commonly termed enteric neuropathies^1–6^. Such enteric neuropathies arise developmentally via disrupted development of the ENS or postnatally via specific neuronal loss or the disturbance of neuronal signalling. Current interventions for the treatment of these diseases are mainly focused on symptom management and are limited to chronic pharmacological treatment, or surgical resection of the affected regions in the most severe cases^2,7^. Unfortunately, in a significant proportion of patients such interventions result in significant morbidity, and poor prognosis^8,9^, with patients often requiring further surgical management through early childhood and adolescence^10–13^. Given the failure of currently available surgical techniques and drug regimens to provide adequate treatment for such conditions, alternative therapeutic approaches are required.

Recently, significant research efforts have been employed to investigate the potential of autologous enteric neural stem cells (ENSC) as a possible treatment option to replace lost or damaged neurons in a range of mouse models. Early proof-of-principle studies have established the potential for in vivo transplantation of ENSC-derived neurons in wild-type^14–16^ and dysmotile transgenic tissues^17,18^. Importantly, these studies have shown the successful long-term engraftment of ENSC and their derivatives within the colonic *muscularis.* Interestingly, donor-derived cells have been observed to engraft and extend processes at the site of transplantation, forming anastomosing networks of donor cells within host tissues, which appear to functionally integrate with the endogenous ENS. Moreover, donor-derived neurons have been observed at considerable distances from the presumptive site of transplantation, and appear able to migrate through the *muscularis* to reside within endogenous ganglia structures at the level of the myenteric plexus.

However, how transplanted cells integrate into host tissues is currently unclear. Here we show, using an ex vivo organotypic culture system, that integration of ENSC-derived cells within myenteric ganglia occurs across a three-week timeframe. We further demonstrate that such integration requires the dynamic remodelling of collagen components within the extracellular matrix (ECM), and tissue architecture, as donor cells migrate across the gut wall. Thus, we propose that these processes are rate limiting factors in the successful functional integration of ENSC-derived neurons within transplanted tissues, and are therefore fundamental considerations in the successful implementation of ENSC-based therapeutic approaches to treat enteric neuropathies.

## Methods

### Animals

Animals were obtained from The Jackson Laboratory (Bar Harbor, MN, USA). For experimental procedures, adult C57BL/6J wild-type (6–8 week old) were used to obtain recipient tissue, and early postnatal (day 5–7) *Wnt1*^*cre/*+^*;R26R*^*YFP/YFP*^ mice (where neural crest cell derivatives express yellow fluorescent protein (YFP)) were used as donors. Animals were housed and experiments were performed in accordance with relevant ARRIVE guidelines, the UK Animals (Scientific Procedures) Act 1986, and approved by the University College London Biological Services Ethical Review Process. Animal husbandry at UCL Biological Services was in accordance with the UK Home Office Certificate of Designation.

### Donor cell isolation and enrichment

Typically, the entire gut (small intestine and colon) was obtained from 2 to 3 *Wnt1*^*cre/*+^*;R26R*^*YFP/YFP*^ mice at P5-7 after cervical dislocation. Tissues were removed to sterile phosphate-buffered saline (PBS, 0.01 mol L^−1^, pH 7.2 at 4 °C) for further dissection. Strips of the *tunica muscularis* were obtained from the jejunum, ileum and colon following removal of the mucosa, via fine dissection, and pooled for enzymatic dissociation.

Single intestinal cells were obtained after enzymatic dissociation of the *tunica muscularis* using a Tumor Dissociation Kit (Miltenyi Biotec, Woking, UK), and YFP^+^ cells isolated using fluorescence activated cell sorting (FACS) with a MoFloXDP cell sorter (Beckman Coulter, Wycombe, UK). YFP^+^ cells were selected using a 530/40 filter set. The ‘n values’ reported refer to the independent FACS experiments.

### Neurosphere culture

YFP^+^ cells were plated at a minimum seeding density of 1 × 10^5^ cells/well on fibronectin-coated (2% w/v in 0.1 mol L^−1^ PBS, Sigma-Aldrich, Gillingham, UK) 6-well dishes. Plated YFP^+^ cells were maintained in “neurosphere medium” (NSM; DMEM/F12 supplemented with B27 (Gibco, Hemel Hempstead, UK), N2 (Gibco), 20 ng/ml epidermal growth factor (EGF, PeproTech, London, UK), 20 ng/ml fibroblast growth factor (FGF, PeproTech), and Primocin (100 μg/ml; InvivoGen, Toulouse, France) antibiotic. Typically, such cultures from P5-P7 intestine formed “neurospheres” between 1 and 2 weeks and were maintained in culture for up to 4 weeks.

### Organotypic culture

The entire colon from adult (6–8-week-old) C57BL/6J mice was removed to sterile PBS (0.01 mol L^−1^, pH 7.2 at 4 °C) after cervical dislocation. Colonic tissues were pinned in a Sylgard-lined chamber and opened along the length of the colon at the mesenteric border. The mucosa was removed, via fine dissection, taking special care to avoid any damage to the underlying *tunica muscularis*. Tissue segments were then mounted (serosa side down) on sterilized 5 mm diameter metal tissue mounts (custom made in-house; UCL Workshop) and fixed in place with sterilized “O-rings” (Fisher Scientific, Loughborough, UK). Tissues were washed thoroughly in sterile PBS (0.01 mol L^−1^, pH 7.2) supplemented with Primocin (100 mg/ml; InvivoGen) and maintained, at 37 °C, 95% O_2_/5% CO_2_, in Dulbecco’s Modified Eagle Medium (DMEM, Gibco) supplemented with L-Glutamine and Primocin (100 mg/ml; InvivoGen) for 1 h before neurosphere transplantation. The ‘n values’ reported refer to the number of colonic segments, each from a separate mouse, examined at each timepoint.

### Ex vivo neurosphere transplantation

Colonic tissue scaffolds were inverted and placed, submucosal side down, into individual wells of an untreated 6-well plate. An individual YFP^+^ neurosphere was subsequently transplanted to the serosal surface, by mouth pipette, using a pulled glass micropipette. Care was taken to gently place the neurosphere in a central position, without disruption of the underlying tissue. 15 µl media (DMEM, L-Glutamine and Primocin) was added inside the scaffold well and transplanted tissues were incubated for 2 h at 37 °C, allowing attachment of the neurosphere to the serosal surface. Subsequently, wells were supplemented with 3 ml media (DMEM, L-Glutamine and Primocin) and were maintained in culture for between 1 and 21 days post-transplantation.

### Immunohistochemistry

Tissues were fixed in paraformaldehyde (4% w/v in 0.1 mol L^−1^ PBS) for 45 min at room temperature (RT). After fixation, tissues were washed thoroughly for 1 h in PBS (0.01 mol L^−1^, pH 7.2 at RT). Tissues were blocked for 1 h (0.1 mol L^−1^ PBS containing 1% Triton X-100, 10% sheep serum). Tissues were incubated in primary antibody (diluted in 0.1 mol L^−1^ PBS containing 1% Triton X-100, 10% sheep serum, Supplementary Table 1) for 16 h at 4 °C and immunoreactivity was detected using the secondary antibodies listed in Supplementary Table 2 (1:500 in 0.1 mol L^−1^ PBS, 1 h at RT). Before mounting, tissues were washed thoroughly in PBS (0.1 mol L^−1^ PBS for 2 h at RT). Control tissues were prepared by omitting primary or secondary antibodies. Tissues were examined using as LSM710 Meta confocal microscope (Zeiss, Munich, Germany). Confocal micrographs were digital composites of Z-series scans (0.5–1 μm optical sections). Each antibody label was assessed in a minimum of three independent replicates to determine reproducibility and consistency. Final representative images were constructed using FIJI software^19^. Depth-coding of confocal metafiles was performed using Imaris Cell Imaging Software (Oxford Instruments, Zurich, Switzerland).

### Quantification of transplanted ENSC spread

For montage experiments of cell spread, tissues were examined using an Axioplan Observer microscope (Zeiss). Micrographs of wholemounts were digital composites of individual 20X tiled micrographs, stitched using Zen software (Zeiss). Final images were constructed using FIJI software. The area of donor cell migration within recipient colonic tissue scaffolds was quantified (Adobe Photoshop, CA, USA), with individual and mean values plotted using GraphPad Prism software (GraphPad, CA, USA). The ‘n values’ reported refer to the number of colonic segments, each from a separate mouse, which received cells from an independent donor cell isolation experiment.

### Quantification of donor cell z-axis integration

To examine integration through the gut wall transplanted tissues were immunohistochemically labelled, as above, including DAPI as standard. Following confocal imaging (1 μm optical sections), maximal *z-axis* integration of donor cell body depth was determined by identifying, and recording, the most medially integrated donor cell body (GFP^+^/DAPI^+^), relative to the serosal surface. The ‘n values’ reported refer to the number of colonic segments, each from a separate mouse, which received cells from an independent donor cell isolation experiment.

### RT-PCR

RNA was extracted from control and transplanted tissues using TRIzol reagent (ThermoFisher, Hemel Hempstead, UK) and treated with DNase I (Qiagen, Manchester, UK). First-strand cDNA was amplified from 100 ng RNA using SuperScript VILO cDNA Synthesis Kit (ThermoFisher). qRT-PCR was performed with a StepOnePlus Real-Time PCR System (ThermoFisher) using the Quantitect SYBR Green PCR kit (Qiagen), according to the manufacturer's instructions. qRT-PCR was performed in triplicate, using region-specific primers designed against mouse sequences for *Gapdh, Col1a, Col4a, Fn, Lama1, Lamb1, Eln, Mmp2, Mmp8, Mmp9 and Mmp13* (Supplementary Table 3). Gene expression data were expressed as a proportion of *Gapdh*, as a reference, using ΔCT and ΔΔCT calculations. The ‘n values’ reported refer to the number of control and transplanted colonic segments, each from a separate mouse. Transplanted segments received cells from independent donor cell isolation experiments.

### Statistical analysis

Data are expressed as mean ± standard error of the mean. Statistical analysis was performed using GraphPad Prism software (GraphPad). The intergroup differences for neural network quantification, and maximal z-axis integration post-transplantation, at day 7, 14, & 21, were evaluated using Welch’s ANOVA and Welch’s t-tests. For transcriptomic analyses statistical comparison between control (non-transplanted) and transplanted samples was performed using ΔCT values by Welch’s t-test. Results were considered significant at *P* < 0.05.

### Ethical approval and consent to participate

The animals used and the experiments conducted, in this study, were approved by the University College London Biological Services Ethical Review Process. All of the procedures performed were in accordance with the UK Animals (Scientific Procedures) Act 1986.

## Results

### Characterisation of ex vivo organotypic cultured colon

**Figure 1:**
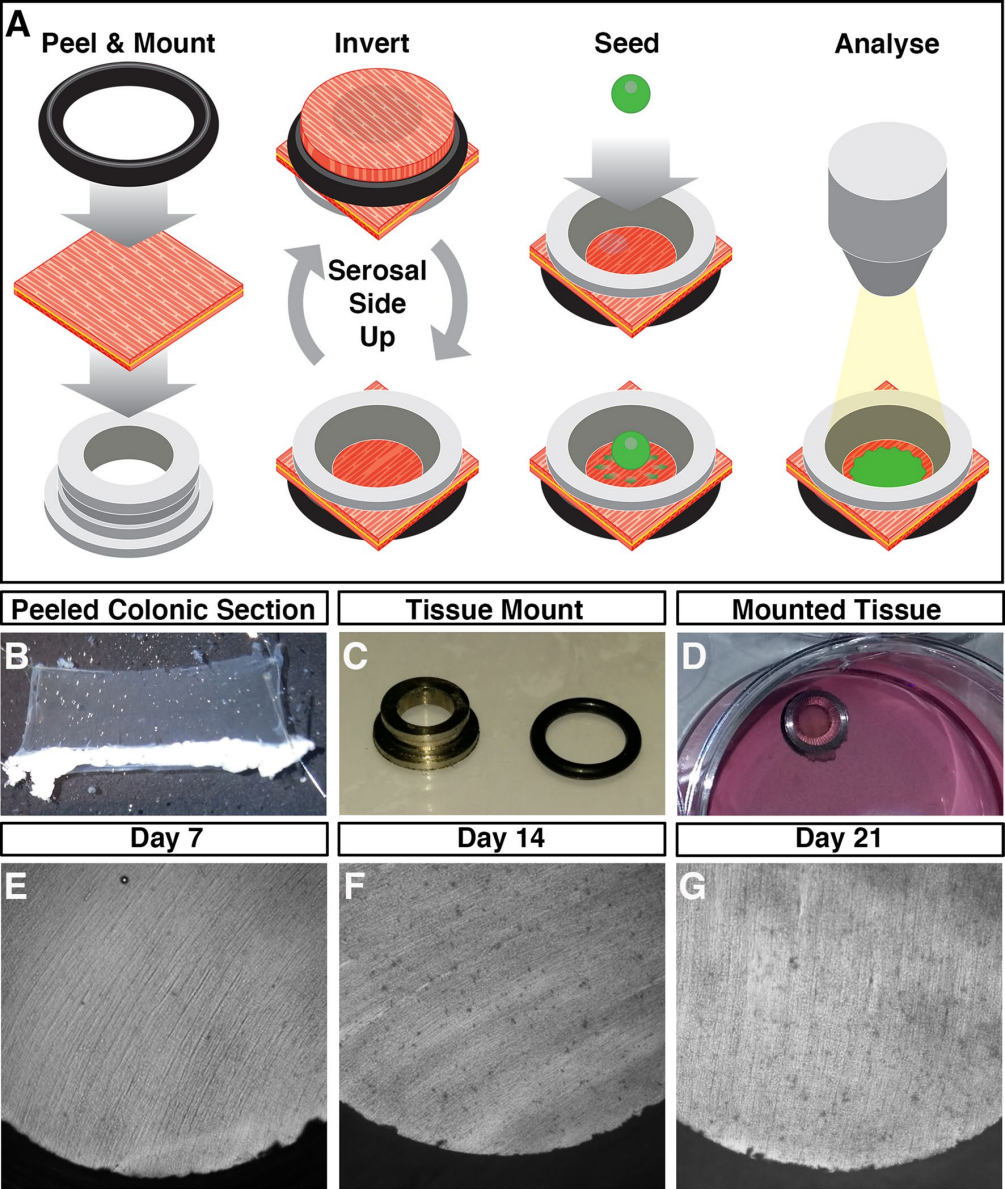
Ex vivo organotypic culture of colonic tissue. (**A**) Schematic representation of ex vivo gut culture methodology and the strategy used to perform ENSC transplantation. Illustration created by UCL Medical Illustration. (**B**) Representative image of colonic *tunica muscularis* after fine dissection of the mucosal layer. (**C**) Representative image of tissue mounting apparatus. (**D**) Representative image of mounted colonic tissue segment in organotypic culture. (**E**–**G**) Representative images of cultured colonic tissues at Day 7 (**E**), Day 14 (**F**), and Day 21 (**G**) demonstrating the absence of contamination, and lack of gross tissue damage, within long-term organotypic cultures.

To assess the integration of ENSC, within intestinal segments, we developed an ex vivo organotypic culture model, based upon methodology previously used for ENS imaging^20,21^, in which colonic tissues could be maintained in situ with limited contractile forces (Fig. 1A–D), which typically lead to tissue damage when pinned. Macroscopic images of tissue segments demonstrate well-preserved, undamaged, tissue structures at day 7, 14 and 21 (Fig. 1E–G). Remarkably, up to 21 days in culture such preparations did not display evidence of culture contamination, visible structural abnormalities or any signs of detrimental tissue-scaffold interaction.

**Figure 2:**
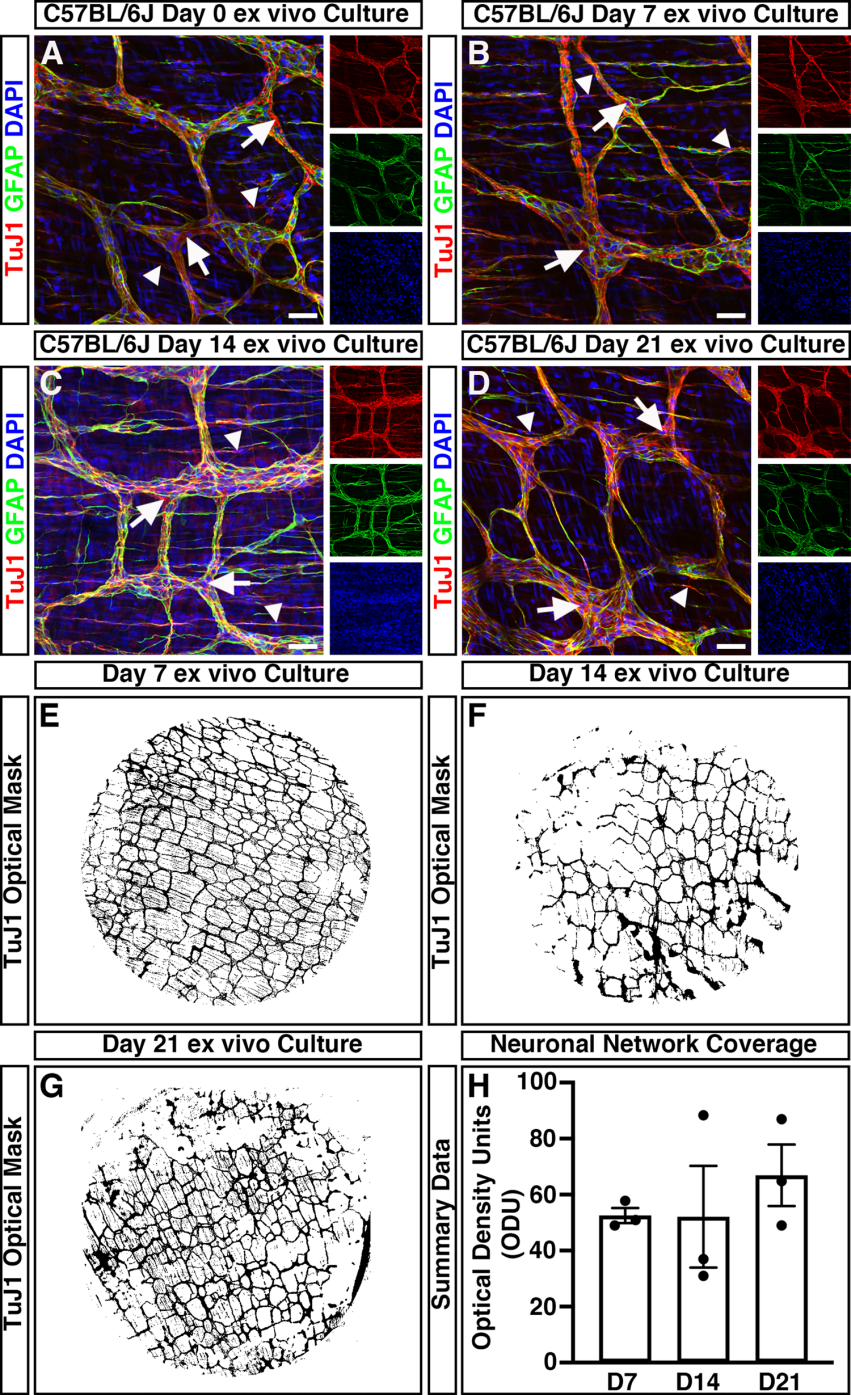
Mouse colonic segments cultured ex vivo maintain expression of ENS markers. (**A**–**D**) Representative confocal z-stack images demonstrating the presence of the pan neuronal marker; TuJ1 (*red*), glia marker; GFAP (*green*) and DAPI (*blue*) in fresh (**A**), Day 7 (**B**), Day 14 (**C**), and Day 21 (**D**) ex vivo cultured C57BL/6J colonic tissue. TuJ1^+^ expression was readily observed across organotypic culture and was found to label the ENS at the level of the myenteric plexus (arrows) and intramuscular nerves (arrowheads). (**E**–**G**) Representative optical density masks of montaged TuJ1^+^ expression within cultured colonic segments at Day 7 (**E**), Day 14 (**F**), and Day 21 (**G**). (**H**) Summary data of neuronal network coverage, as determined by the optical density of TuJ1^+^ expression, across organotypic culture (n = 3 for each timepoint). Error bars represent mean ± s.e.m. Scale bars represent 50 μm.

To assess the impact of organotypic culture on the development and maturation of the ENS, immunohistochemistry was performed. Within freshly dispersed (uncultured) colonic tissues, robust neuron-specific class III beta-tubulin positive (TuJ1^+^) neuronal networks could be observed (Fig. 2A). TuJ1^+^ neuronal networks were observed, organised in a characteristic mesh-like structure, at the level of the myenteric plexus, in close apposition to glial fibrillary acidic protein positive (GFAP^+^) glial cells (Fig. 2A, arrows). Additional TuJ1^+^ intramuscular neurons were observed extending bipolar neuronal fibres within the *tunica muscularis* (Fig. 2a, arrowheads). Notably, organotypically cultured tissues demonstrated similar TuJ1^+^ and GFAP^+^ expression at day 7 (D7; Fig. 2B), day 14 (D14; Fig. 2C), and day 21 (D21; Fig. 2D) in culture. Again, Tuj1^+^ neuronal populations could be observed both at the level of the myenteric plexus (arrows) and intramuscularly (arrowheads).

To quantitatively assess the condition of the endogenous enteric neural network, and to define its structure over time in organotypic culture, the optical density of TuJ1^+^ positive pixels within wholemount montages of cultured colonic specimens was assessed. The mean values of the optical density of TuJ1^+^ neuronal networks between D7 (52.5 ± 2.7 optical density units (ODU); n = 3, Fig. 2E, H), D14 (52.1 ± 18.2 ODU; n = 3; Fig. 2F,H) and D21 (66.9 ± 11.0 ODU; n = 3; Fig. 2G,H) cultures where comparable (W_2.0, 2.9_ = 0.66, *P* = 0.582) as determined by Welch’s one way analysis of variance (ANOVA). These data suggest the preservation of neuronal networks within ex vivo organotypically cultured specimens up to 21 days in culture.

### Donor ENSC migrate extensively within organotypic cultures

**Figure 3:**
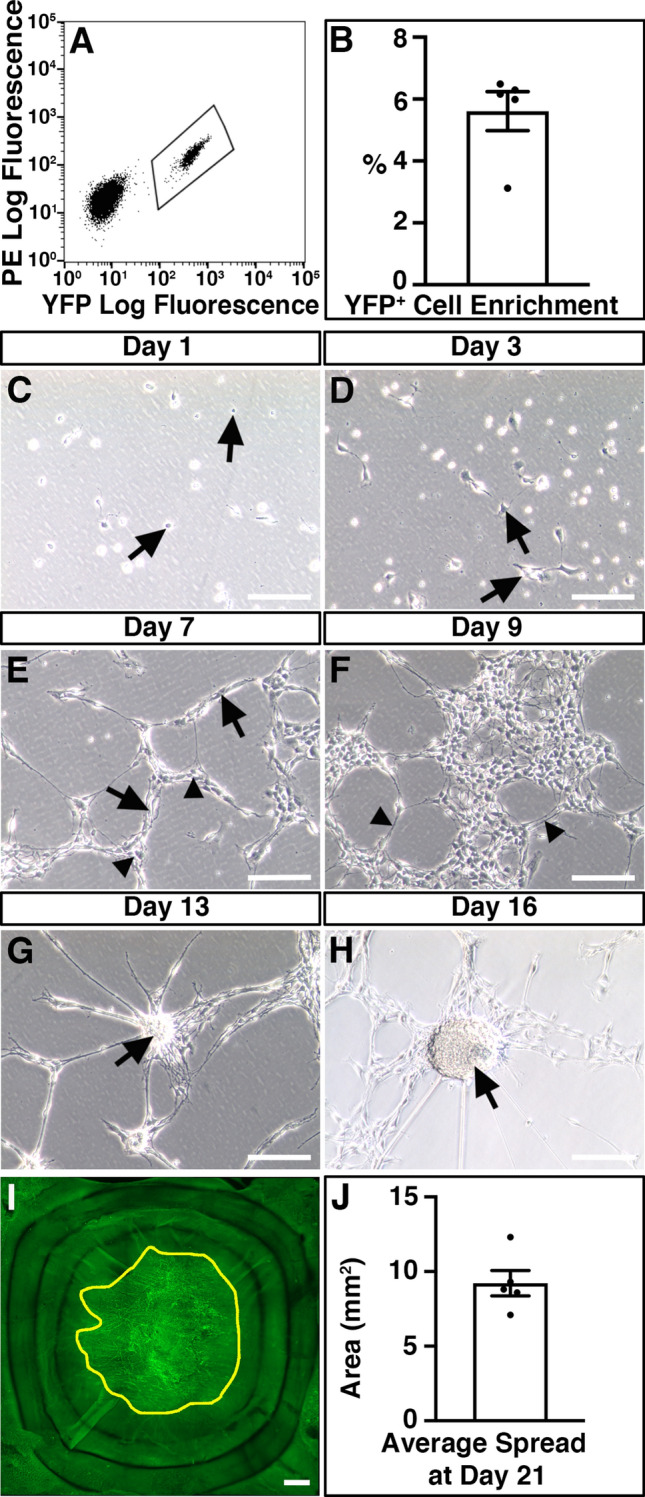
Isolation and expansion of donor ENSC for ex vivo transplantation. (**A**) Representative FACS profile demonstrating the gating scheme for isolation of YFP^+^ cells (bounded area) from *Wnt1*^*cre/*+^*;R26R*^*YFP/YFP*^ intestine. (**B**) Summary data showing YFP^+^ cell enrichment via FACS (n = 5). (**C**–**H**) Representative images showing the development of FACS-sorted YFP^+^ cells in culture. At Day 1 (**C**), the majority of sorted YFP^+^ cells displayed a spherical appearance (arrows). By Day 3 (**D**), YFP^+^ cells developed a typical multipolar neural crest cell appearance (arrows). At Day 7 in culture (**E**), sorted cells appear as multipolar cell clusters (arrows) which are connected in a network-like structure (arrowheads). At Day 9 (**F**), YFP^+^ cell clusters appeared to form extensive networks which displayed interconnecting filaments (arrowheads). Further culture to Day 13 (**G**) and Day 16 (**H**) led to the development of rounded “neurosphere” structures (arrows). (**I**) Representative montage fluorescent image showing the development of YFP^+^ donor cells within recipient C57BL/6J colonic tissue 21-days after ex vivo transplantation. Yellow line bounds YFP^+^ donor cell coverage. (**J**) Summary data showing average donor cell spread within recipient tissues at Day 21 (n = 5). Error bars represent mean ± s.e.m (**B**, **J**). Scale bars represent 250 μm (**C**–**H**), 50 μm (**I**). See also Supplementary Fig. 1.

ENSC were isolated from donor *Wnt1*^*cre/*+^*;R26R*^*YFP/YFP*^ mice (P5-P7), in which neural crest cells and their derivatives express endogenous yellow fluorescent protein (YFP). This endogenous YFP expression allowed isolation, by FACS, and fate-labelling of donor ENSC (Fig. 3A,B). Typically, enzymatic digestion of the *tunica muscularis,* from the entire small intestine and colon, and subsequent FACS lead to the enrichment of a YFP^+^ neural crest-derived population at 5.6 ± 0.63% (3.2 × 10^5^ ± 5 × 10^4^ cells) per sorting experiment (Fig. 3B; n = 5). Upon culture, individually sorted YFP^+^ cells appeared as sparsely distributed spherical shaped cells (Fig. 3C, arrows). By D3, selected YFP^+^ cells developed a typical multipolar neural crest cell appearance and began to form cellular connections (Fig. 3D, arrows). At D7, cell clusters (Fig. 3E, arrows) and elongated interconnected “chains” of cells could be observed forming a network-like structure (Fig. 3E, arrowheads). Further culture to D9 resulted in the expansion of YFP^+^ ENSC cell clusters to form extensive networks which displayed interconnecting filaments (Fig. 3F, arrowheads). Subsequently, selected YFP^+^ ENSC-derived cells were found to form multiple characteristic three dimensional “neurospheres” by approximately 2 weeks in culture (Fig. 3G,H, arrows).

In order to assess integration of ENSC-derived cells, in intestinal specimens, we sought to establish the ability of cultured neurospheres to colonise and integrate, within organotypically cultured C57BL/6J colonic specimens ex vivo.

We transplanted a single, individual YFP^+^ neurosphere (approximately 2 × 10^4^ cells) to the serosal surface of organotypically cultured C57BL/6J colonic specimens (Supplementary Fig. 1A). At day one (D1) in culture, neurospheres appeared to be attached to recipient C57BL/6J colonic sections and multiple YFP^+^ cells were found to have dissociated from the presumptive site of transplantation, which were observed migrating as individual cells at the periphery of the donor neurosphere (Supplementary Fig. 1B). At D3, migration of donor cells was observed to continue in a non-uniform fashion in all directions away from the transplantation site (Supplementary Fig. 2). After three weeks (D21) in culture, neurosphere-derived donor YFP^+^ cells were found to have migrated extensively to cover an average area of 9.22mm^2^ ± 0.85mm^2^ (approximately 47% ± 4%) within recipient colonic tissues (Figs. 3I,J).

### Temporal integration of ENSC-derived cells within organotypic cultures

To assess integration of YFP^+^ donor cells, within the *tunica muscularis* of recipient colonic segments, immunohistochemistry was performed using the pan-neuronal marker TuJ1 and green fluorescent protein (GFP) antibody.

**Figure 4:**
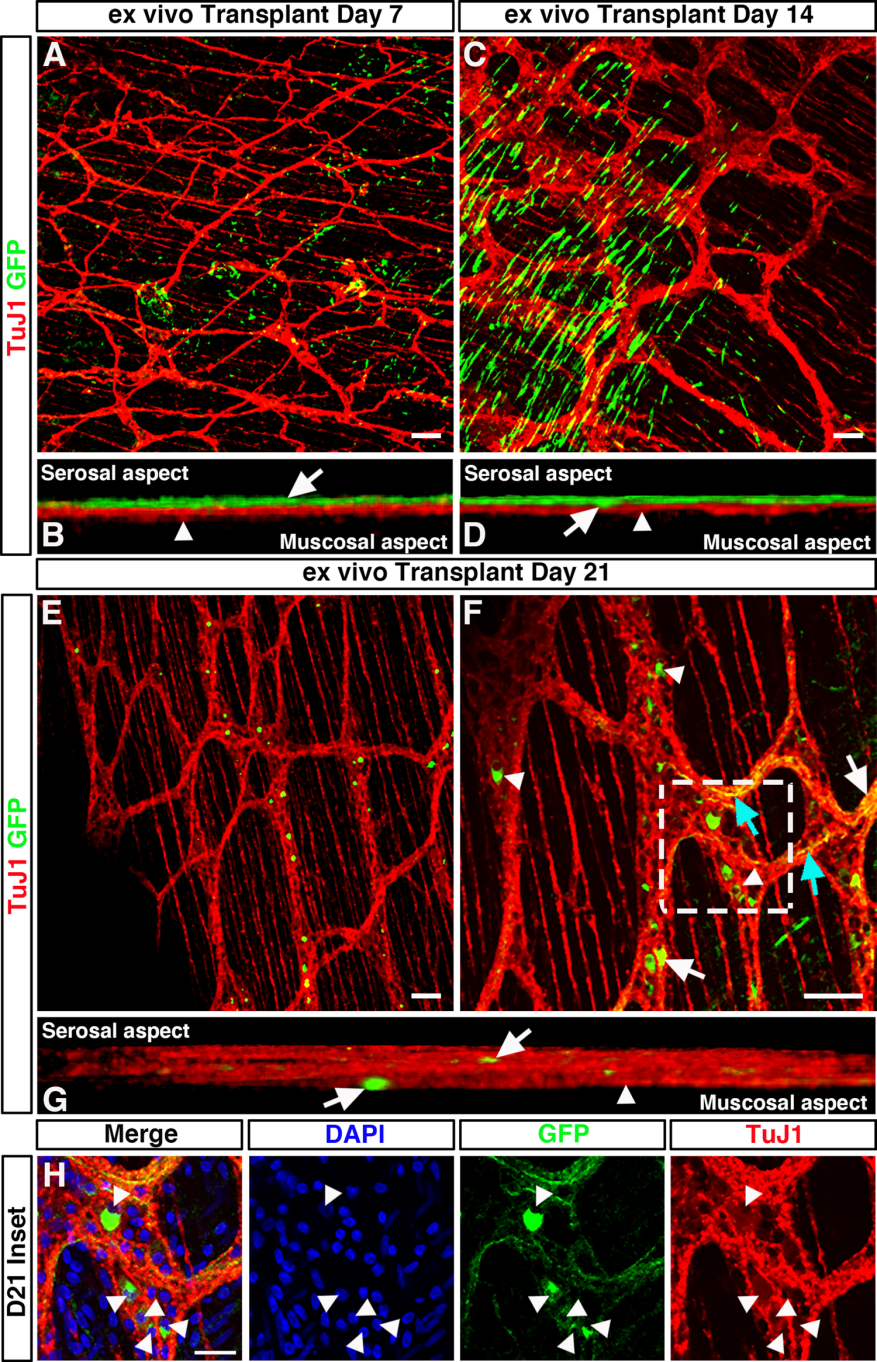
Temporal integration of transplanted ENSC within ex vivo organotypically cultured C57BL/6J colon. (**A**) Representative confocal z-stack image of GFP^+^ (*green*) ENSC-derived cells and the endogenous TuJ1^+^ (*red*) ENS 7-days after ex vivo transplantation. (**B**) Orthogonal view of 3D rendered confocal stack showing donor GFP^+^ cells at the serosal aspect (*arrow)* and TuJ1^+^ myenteric plexus (arrowhead) 7-days after ex vivo transplantation. (**C**) Representative confocal z-stack image of donor GFP^+^ (*green*) cells and the endogenous TuJ1^+^ (*red*) ENS 7-days after ex vivo transplantation. (**D**) Orthogonal view of 3D rendered confocal stack, 14-days after ex vivo transplantation, showing donor GFP^+^ cell penetration (*arrow*) from the serosal aspect towards the myenteric plexus (arrowhead). (**E**, **F**) Representative low- (**E**), and high-power (**F**) confocal z-stacked images of GFP^+^ (*green*) donor cells within the recipient TuJ1^+^ (*red*) myenteric plexus, 21-days after ex vivo transplantation. Donor cells were observed as both individual cells, and donor cell clusters, within ganglia like structures (arrowheads). Donor derived GFP^+^TuJ1^+^ neurons (arrows) appeared to extend GFP^+^TuJ1^+^ neuronal fibres which traced the endogenous neural network (cyan arrows). (**G**) Orthogonal view of 3D rendered confocal stack showing donor GFP^+^ cells (*arrows)* within the endogenous TuJ1^+^ myenteric plexus (arrowhead) 21-days after ex vivo transplantation. (**H**) Zoomed image of inset shown in (**F**) showing high magnification of donor cell bodies (arrowheads) incorporated within ganglia as indicated by DAPI (*blue*), GFP (*green*) and TuJ1 (*red*). Scale bars represent 50 μm (**A**–**G**), 25 μm (**H**).

At D7, GFP^+^ cells were observed on the serosal aspect of transplanted colonic segments and appeared to migrate in multiple directions (Fig. 4A). At this stage, no penetration or integration of donor GFP^+^ cells, within the *tunica muscularis*, was observed. Donor cells were observed on the serosal aspect only (Fig. 4B; arrow), with no observable migration toward the endogenous TuJ1^+^ ENS at the level of the myenteric plexus (Fig. 4B; arrowhead).

At post-transplantation D14, transplanted YFP^+^ neurospheres appeared to have dissociated into single cells (Fig. 4C) with numerous, spindle shaped, GFP^+^ cells observed. The majority of GFP^+^ cells at D14 were again observed at the outer serosal aspect of transplanted tissues. However, donor GFP^+^ cells were observed which displayed a limited degree of tissue penetration (Fig. 4D; arrow) towards the myenteric plexus (Fig. 4D; arrowhead).

At D21, transplanted GFP^+^ ENSC-derived cells were visualised at the serosal aspect (Supplementary Fig. 3A). Though by contrast, GFP^+^ donor cells were also observed to have migrated through the muscularis to integrate within, or in close association with, recipient endogenous enteric ganglia at the level of the myenteric plexus (Fig. 4E–H, Supplementary Fig. 3B). GFP^+^ cells were observed distributed along the “mesh-like” myenteric neural network, both as individual cells, and as donor cell clusters, within individual ganglia structures (Fig. 4F–H). At this timepoint, donor derived GFP^+^ cells could be observed having differentiated to a neuronal phenotype with co-expression of either TuJ1 (Fig. 4F; arrows) or HuC/D (Supplementary Fig. 3B, Supplementary Movie 1). Moreover, GFP^+^TuJ1^+^ neuronal fibres were also observed at the level of the myenteric plexus, which appeared to trace the endogenous recipient neural network (Fig. 4F; cyan arrows). Upon three-dimensional (3D) reconstruction, donor GFP^+^ cells could clearly be observed throughout the TuJ1^+^ myenteric neural network (Fig. 4G; arrowhead), including at both the serosal and mucosal aspects of the myenteric plexus (Fig. 4G; arrows). Here, individual donor-derived GFP^+^ cell bodies could be observed within endogenous ganglia structures, via visualisation of DAPI expression within GFP^+^ donor cells (Fig. 4H, arrowheads).

To establish if donor cells undergo proliferation and/or apoptosis, upon transplantation, we assessed Ki67 & cleaved Caspase 3 expression in neurospheres and transplanted ex vivo tissues at early (D3) and late (D21) timepoints (Supplementary Figs. 4, 5). Proliferation and apoptosis of *Wnt1*^*cre/*+^*;R26R*^*YFP/YFP*^-derived cells were clearly observed pre-transplantation, within equivalent neurospheres, as evidenced by robust expression of both Ki67 (Supplementary Fig. 4A) and cleaved Caspase 3 (Supplementary Fig. 5A). However, shortly after transplantation (D3), neither Ki67 (Supplementary Fig. 4B) nor cleaved Caspase 3 (Supplementary Fig. 5B) were observed within GFP^+^ donor cells. Similarly, at D21 post-transplantation, we were unable to observe GFP^+^-donor derived cells which were undergoing proliferation, or apoptosis, despite evidence of Ki67^+^ and Caspase3^+^ expression in the recipient *muscularis* (Supplementary Figs. 4C, 5C).

**Figure 5:**
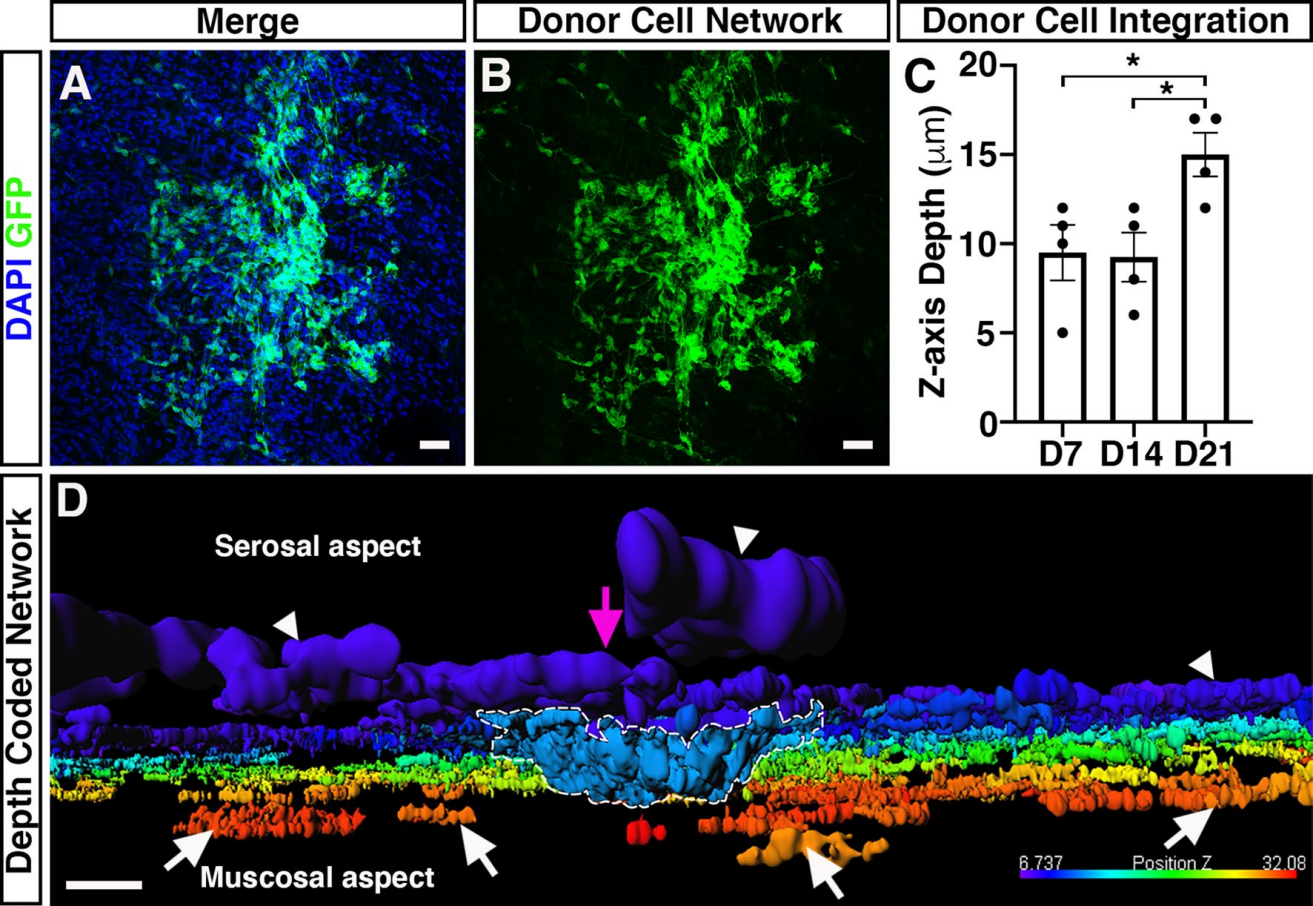
Integration of ENSC-derived donor cells across the gut wall. (**A**, **B**) Representative confocal z-stack image of donor ENSC-derived GFP^+^ (*green*), and DAPI (blue), within the gut wall 21-days after ex vivo transplantation. (**C**) Summary data showing analysis of donor cell body (GFP^+^/DAPI^+^) integration, measured as z-axis depth, across the gut wall at day-7 (D7), D14, and D21 after ex vivo transplantation (n = 4 for each timepoint). (**D**) Representative depth-coded image of donor cell integration at D21. Note the protrusion of donor ENSC-derived cells into the gut wall (*dashed line*) from the site of transplantation on the serosal surface (*magenta arrowhead*). Donor GFP^+^ cells and fibres were observed to have integrated longitudinally along the gut, both on the serosal surface (*arrowheads*) and within the *tunica muscularis* (*arrows)*. Scale bars represent 50 μm (**A**, **B**), 30 μm (**D**). Error bars represent mean ± s.e.m. *P ≤ 0.05, by Welch’s t-test.

We next aimed to quantify the maximal distance ENSC-derived cells had migrated across the gut wall using confocal z-stacks of GFP-labelled donor cells, in combination with DAPI labelling (Fig. 5A, B). Here, maximal cell integration was recorded by observing the most medially integrated donor cell body (GFP^+^/DAPI^+^), relative to the serosal surface using Z-series scans (1 μm optical sections), which showed significant differences in the mean values across the three timepoints as determined by Welch’s ANOVA (W_2.0, 5.9_ = 5.58, *P* = 0.04). At D7, ENSC-derived donor cells were observed at a z-axis depth of 9.5 ± 1.6 μm (Fig. 5C; n = 4). Similarly, at D14 donor-derived cells were observed at a z-axis depth of 9.3 ± 1.4 μm (*P* = 0.91; n = 4; Fig. 5C). However, at D21 the most medially integrated donor-derived cell body in ex vivo cultured transplanted colon was observed at a z-axis depth of 15 ± 1.2 μm (*P* = 0.03 vs D7, *P* = 0.02 vs D14; n = 4; Fig. 5C). Importantly, depth-coding of all GFP^+^ donor-derived cell bodies and fibres, according to their z-axis depth, revealed integration of donor ENSC along the serosal aspect of the gut (Fig. 5D; arrowheads, Supplementary Movie 1). Moreover, from the presumptive site of transplantation (Fig. 5D; magenta arrow), donor cells appear to penetrate the gut wall at the site of neurosphere engraftment (Fig. 5D; dashed white line) and migrate along the longitudinal aspect of the gut within the *tunica muscularis* (Fig. 5D; white arrows).

Taken together, these data suggest that ENSC appear to initially migrate from the transplanted neurosphere, along the serosal aspect of the tissue, in a non-uniform manner. Subsequently, transplanted cells penetrate the *tunica muscularis* at the site of neurosphere transplantation and migrate within the *tunica muscularis* towards the endogenous neural network at the level of the myenteric plexus, allowing integration within ganglia structures and extension of donor cell processes, within the myenteric network, by approximately three weeks.

### Molecular characterisation of donor ENSC integration after transplantation

Having demonstrated the integration of donor ENSC-derived cells, within the *tunica muscularis,* we assessed possible tissue remodelling within the gut wall; and the molecular basis of ENSC migration and integration within transplanted colonic segments.

**Figure 6:**
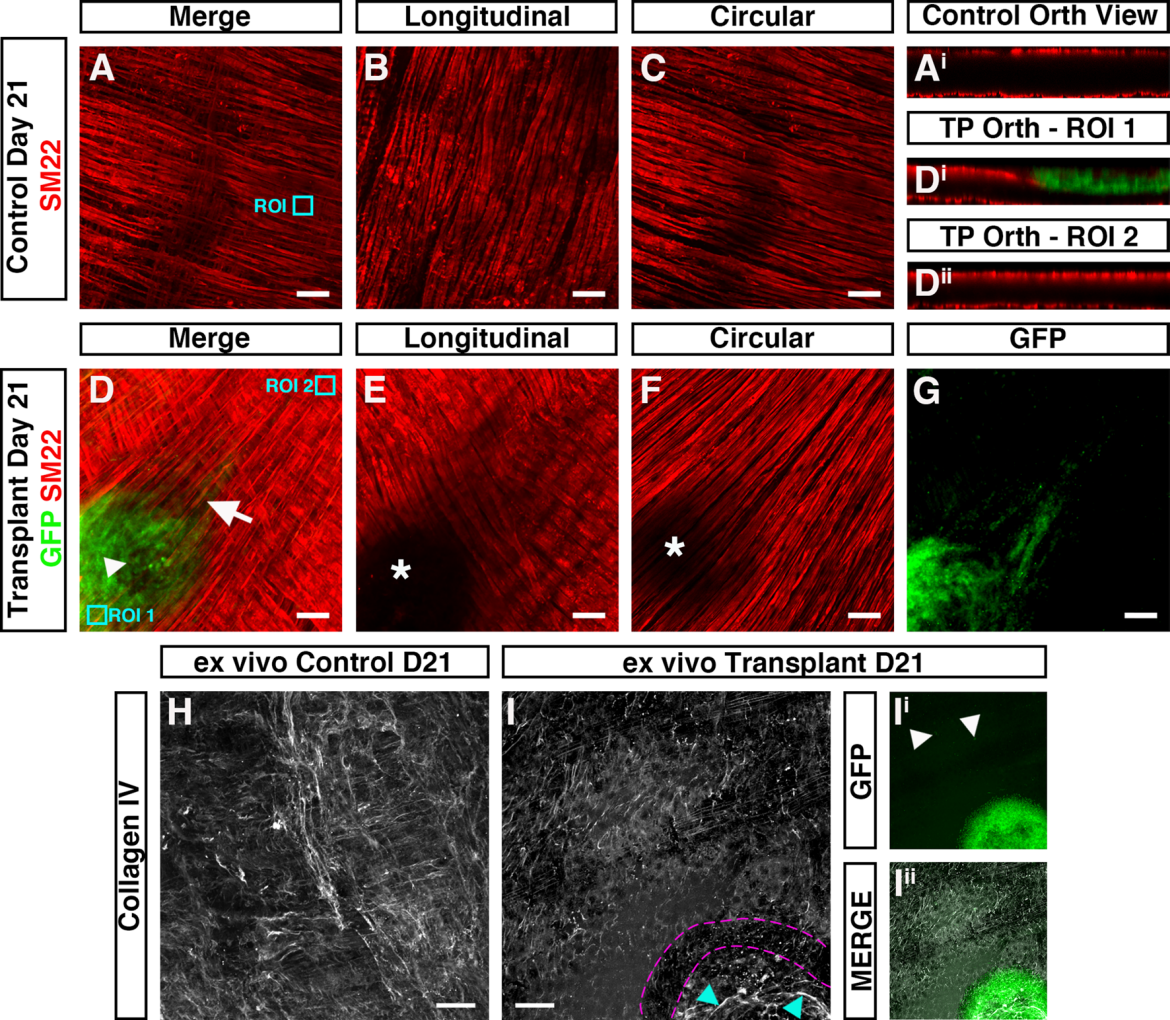
Donor cell integration induces tissue remodeling after ex vivo transplantation. (**A**) Representative confocal z-stack image of SM22^+^ (*red*) smooth muscle within control (non-transplanted) C57BL/6J colonic tissue at day-21. (**A**^**i**^) Orthogonal view taken from the region of interest (*ROI, cyan box)* in *A* showing SM22^+^ smooth muscle in two uninterrupted layers. (**B**, **C**) Representative images of individual z-stacks at the longitudinal (**B**) and circular (**C**) muscle layers. (**D**) Representative confocal z-stack image of donor GFP^+^ (*green*) and SM22^+^ (*red*) smooth muscle 21-days after ex vivo transplantation. (**D**^**i**^**&D**^**ii**^) Orthogonal views taken from *ROIs (cyan boxes)* in (**D**) showing interruption of the longitudinal smooth muscle (**D**^**i**^) at the site of neurosphere engraftment. At sites away from the area of neurosphere engraftment SM22^+^ expression appears to demonstrate two uninterrupted smooth muscle layers (**D**^**ii**^). (**E**, **F**) Representative images of individual z-stacks at the longitudinal (**E**) and circular (**F**) muscle layers 21-days after ex vivo transplantation. (**G**) Representative image of individual z-stack, at the level of the circular muscle (taken from **D**), showing migration of GFP^+^ donor derived cells from the site of neurosphere engraftment 21-days after ex vivo transplantation. (**H**) Representative confocal z-stack image of Collagen IV (*grey*) in control (non-transplanted) C57BL/6J colonic tissue at day-21. (**I**) Representative confocal z-stack image of Collagen IV (*grey*) 21-days following ex vivo transplantation. Note the presence of Collagen IV expression at the site of neurosphere transplantation (*cyan arrows*) and apparent discontinuity of Collagen IV expression surrounding the presumptive neurosphere boundary (*magenta dashed lines*). (**I**^**i**^, **I**^**ii**^) Representative GFP channel (**I**^**i**^) and merged (**I**^**ii**^) confocal z-stack images showing GFP^+^ donor cells at the site of transplantation and within the *tunica muscularis* (arrows) having migrated away from the engrafted neurosphere.

In ex vivo control (non-transplanted) tissues, the smooth muscle architecture appears to be preserved up to three weeks in organotypic culture (Fig. 6A–C). Here, immunohistochemistry for the smooth muscle marker SM22 revealed smooth muscle cells in close apposition, both in the longitudinal (Fig. 6B) and circular (Fig. 6C) orientations. Orthogonal reconstruction of confocal z-stacks also revealed intact SM22^+^ muscle layers in both the longitudinal and circular planes (Fig. 6A^i^). By contrast, in transplanted tissues, disruption of the smooth muscle architecture was observed at the site of transplantation 21-days following ENSC transplantation (Fig. 6D–G). Examination of the longitudinal muscle layers revealed complete loss of SM22^+^ expression at the site of neurosphere engraftment (Fig. 6E; white star). Similarly, examination of the circular muscle layer revealed partial reductions in SM22 expression (Fig. 6F; white star). Interestingly, this disruption in SM22 expression appeared to be limited to the site of transplantation and neurosphere engraftment. Upon orthogonal reconstruction of the engraftment site (Fig. 6D, ROI 1), GFP^+^ donor cell expression was observed within the plane of the longitudinal muscle layer (Fig. 6D^i^). While this donor cell expression appeared restricted to the serosal aspect, and presumptive longitudinal muscle layer, all SM22^+^ expression in this engraftment region was absent. However, in regions away from the site of engraftment (Fig. 6D, ROI 2) uninterrupted SM22^+^ expression was observed in both the longitudinal and circular muscle layers (Fig. 6D^ii^). Comparable disruption of the smooth muscle architecture was also observed following assessment of smooth muscle actin expression in independent replicates (Supplementary Fig. 6). Immunohistochemistry also revealed apparent alterations in the collagen ECM compartment within transplanted tissues. As opposed to control non-transplanted colon, where Collagen type IV (Col IV) expression was observed in near continuity (Fig. 6H), loss of Collagen IV was observed at the boundary of the engraftment site in transplanted tissues (Fig. 6I; *magenta border*). Additionally, the presence of Collagen IV expression was observed, at the site of engraftment, within the remaining neurosphere structure (Fig. 6I; *cyan arrow,* Figs. 6I^i^–I^ii^).

To further examine the mechanisms involved in donor cell integration, molecular analyses were performed to examine the expression of candidate genes, known to play critical roles within the intestinal ECM, such as Collagen 1a2 (*Col1a*), Collagen 4a1 (*Col4a*), Fibronectin 1 (*Fn1),* Elastin *(Eln),* Laminin a1 *(Lama1),* Laminin b1 *(Lamb1)* and in remodelling of the ECM, including Matrix metalloproteinase 1 (*Mmp1*), *Mmp2*, *Mmp8, Mmp9* and *Mmp13*. Of note, *Mmp1* could not be resolved in control post-natal C57BL/6J wild type colonic tissue nor in ex vivo cultured control and transplanted tissues whereas all other targets were found to be robustly expressed (*data not shown*), in at least one condition, using standard real-time polymerase chain reaction (RT-PCR) analysis. Interestingly, *Mmp8* was not observed in control postnatal tissues but could be observed qualitatively at very low levels in control ex vivo cultures with increasing expression after transplantation (*data not shown*).

Therefore, all candidate genes, except for *Mmp1*, were taken forward for quantitative RT-PCR (qRT-PCR) analysis to assess their expression in transplanted ex vivo tissue segments compared to control non-transplanted colon.

**Figure 7:**
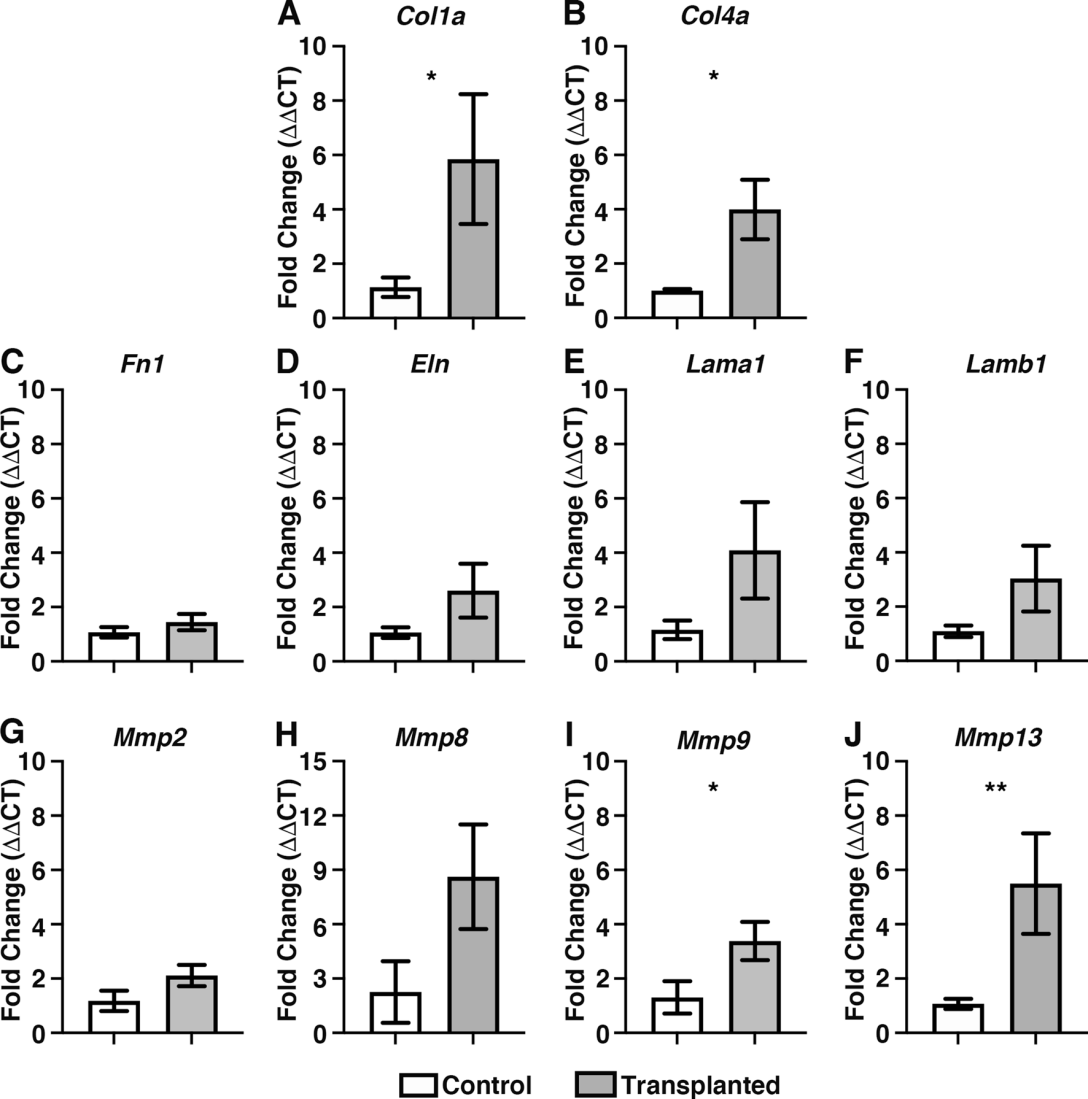
Donor cell integration leads to increased expression of collagen and Matrix metalloproteinases. (**A**–**F**) Summary data showing mRNA fold change for ECM-related and matrix metalloproteinase genes: (*grey bars*): Collagen 1a (*Col1a*; **A**), Collagen 4a (*Col4a*; **B**) Fibronectin 1 (*Fn1*; **C**), Elastin (*Eln*; **D**), Laminin a1 (*Lama1:* **E**), Laminin b1 (*Lamb1*: **F**), Matrix metalloproteinase-2 (*Mmp2*; **G**), Matrix metalloproteinase-8 (*Mmp8*; **H**), Matrix metalloproteinase-9 (*Mmp9*; **I**) and Matrix metalloproteinase-13 (*Mmp13*; **J**) 21-days after ex vivo transplantation compared to non-transplanted control (*white bars*) tissues. Error bars represent mean ± s.e.m. n = 4 for each gene examined. *P ≤ 0.05, **P ≤ 0.01 comparing ΔCT values by Welch’s t-test.

Interestingly, ENSC integration within transplanted tissues led to increased expression of extracellular matrix related genes including *Col1a* (5.8 ± 2.4-fold increase; *P* = 0.04; n = 4; Fig. 7A) and *Col4a* (4.0 ± 1.1-fold increase; *P* = 0.010; n = 4; Fig. 7B) as determined by comparison of ΔCT values by Welch’s t-test analysis. By contrast, other ECM related genes were found to be comparable to control ex vivo cultured tissues: including *Fn1* (*P* = 0.38; n = 4; Fig. 7C), *Eln* (*P* = 0.11; n = 4; Fig. 7D), *Lama1* (*P* = 0.12; n = 4, Fig. 7E) and *Lamb1* (*P* = 0.07; n = 4, Fig. 7F). Of the candidate matrix metalloproteinases, *Mmp2* (*P* = 0.14; n = 4; Fig. 7G) and *Mmp8* (*P* = 0.05; n = 4; Fig. 7H) were found to be expressed at a similar level to control (non-transplanted) tissues; whereas increases in expression were observed in *Mmp9* (3.4 ± 0.7-fold increase; *P* = 0.047; n = 4; Fig. 7I) and *Mmp13* (5.5 ± 1.9-fold increase; *P* = 0.005; n = 4; Fig. 7J) in transplanted tissues when compared to control (non-transplanted) tissues.

Furthermore, assessment of *Mmp* gene expression of *Wnt1*^*cre/*+^*;R26R*^*YFP/YFP*^ neurospheres, by RT-PCR, revealed the expression of *Mmp2* and *Mmp9*. However, *Mmp8* and *Mmp13* could not be identified (Supplementary Fig. 7). Taken together, these data suggest that active remodelling of the ECM collagen compartment and smooth muscle architecture, by both donor-derived and endogenous matrix metalloproteinases, occurs in response to ENSC transplantation, which subsequently allows integration of donor ENSC within and along the gut wall.

## Discussion

Over the past decade there has been an increasing focus on the development and evaluation of stem cell-based therapies for treating enteric neuropathies. Recent studies have highlighted the potential of autologous, and pluripotent-derived ENS progenitor-based therapy, as a means of replacing neurons after in vivo transplantation to mouse colon^14–17,22–26^. However, the precise mechanisms by which donor-derived cells integrate within recipient tissue remain unclear. Therefore, studies to uncover the timeframe, and mechanisms, underlying donor cell integration are fundamental to progressing towards clinical application of stem cell therapy for gut disorders. Moreover, mechanistic studies of donor cell integration may uncover useful targets to improve the efficiency of donor cell integration, and function, within recipient tissues.

Importantly, many previous studies have relied heavily on in vivo surgical transplantation procedures to rodents^14–18,23,27^. While this has provided crucial proof-of-principle data that donor cells *can* integrate within the gut after transplantation, technical limitations such as tissue opacity, and an inability to perform long-term in vivo imaging, have limited the mechanistic investigation of *how* donor cells integrate within recipient gut tissue. Alternative approaches including explant cultures have also established the feasibility of transplanting donor enteric precursors into aneural gut^28–31^. However, these explant methods are often hindered by limitations in their reproducibility, or in the long-term maintenance of tissues on chick chorioallantoic membrane (CAM) cultures^31–33^.

To begin to overcome some of these limitations, here we have employed an ex vivo organotypic culture method to investigate the temporal integration of ENSC within murine gut segments. We demonstrate that this ex vivo organotypic culture configuration allows for long-term culture of murine colonic segments, with preservation of both tissue architecture and ENS structure, providing a robust, reproducible and readily scalable model. Significantly, this method also appears to limit tissue damage, within culture colonic segments, when compared to standard ex vivo organotypic culture conditions which typically require pinning of tissue. Hence, this organotypic method provides an optimal model to explore the temporal integration of donor cells within gut tissues.

Previous studies have detailed the functional integration of donor neurons, derived from postnatally harvested ENSC, in recipient wild-type colonic tissues^14,23^. These studies, using calcium imaging and optogenetic approaches, have detailed the functional integration of donor ENSC-derived neurons within the host neuromusculature four weeks after in vivo transplantation. By four weeks, engrafted cells were found to have migrated extensively within the recipient gut wall, including the integration of donor-derived cells within endogenous ganglia, and the extension of graft-derived axonal fibres which appeared to both trace the endogenous ENS structure, and project to the circular muscle layer. Moreover, engrafted donor-derived neurons were found to both functionally integrate with the endogenous ENS circuitry^14^ and provide inhibitory, and excitatory, innervation to intestinal smooth muscle^23^. A similar timescale was also observed in the functional integration of ENSC-derived neurons within dysmotile transgenic tissues^17^. Here, ENSC transplantation was found to restore neural responses and rescue gut transit four weeks after transplantation. Again, significant migration of ENSC-derived cells was observed both within, and along, the gut wall with donor cells appearing to “home” to the myenteric plexus. More recently, derivation of ENS progenitors from pluripotent sources, such as embryonic stem cells or induced pluripotent stem cells has been proposed as a potential “off-the-shelf” alternative to autologous ENSC^24^. Similar to ENSC, pluripotent-derived ENS progenitors have been shown to engraft and migrate through the colonic *muscularis* to reside within endogenous ganglia structures, across a four week period, following in vivo transplantation^26^. Taken together, these findings suggest that the migration of donor ENSC-derived cells into the gut neuromusculature, and the “homing” of donor neurons to the myenteric plexus region, are likely central processes in the establishment of donor-derived functional responses within the host neuromusculature. However, to date, a more definitive timeframe and mechanistic understanding of donor cell integration remain elusive.

Using our modified ex vivo transplantation approach, we demonstrate that migration of ENSC-derived cells away from the presumptive site of transplantation, and integration within the colonic musculature, is a dynamic process which occurs over a three-week period. Initially, ENSC-derived donor cells were observed to migrate away from the site of neurosphere engraftment, along the serosal aspect of the colon. This migration at the serosal surface did not appear to show preferential directionality and is reminiscent of the migration pattern of cultured neural crest cells^34–36^. A secondary phase of medial migration appears to occur allowing donor cells to integrate across the gut wall. Crucially, this secondary process appears to be established over a number of weeks, with penetration of the neuromusculature, and medial migration, appearing to be prominent only after approximately 14 days.

We hypothesised that donor ENSC utilise molecular processes analogous to that of metastatic cancer cells, in order to remodel the ECM, infiltrate (i.e., intra/extravasate) and migrate (i.e., invade) within the host gut wall following transplantation. Indeed, previous studies have highlighted that the matrisome plays multiple dynamic roles in embryonic neural crest cell migration and differentiation^36–39^. In tandem, migratory neural crest cells have been shown to modify the local ECM, suggesting that migration through the ECM is regulated on multiple levels^40,41^.

Our initial expectation was that migration through the colonic neuromusculature, and integration in myenteric ganglia, would necessitate collagenase-based depletion of the ECM. Our finding of an upregulation in *Mmp9* and *Mmp13* expression, within transplanted ex vivo cultured tissues, fits with our hypothesis as both have been shown to be involved in epithelial-mesenchymal transition (EMT) and the progression of colorectal cancer^42–44^. Importantly, MMP proteolysis of Collagen IV has previously been shown to promote cell migration, via the production of specific cleavage fragments with independent biological activity^45,46^. Therefore, upregulation of *Mmp9* and *Mmp13* in transplanted ex vivo colon may provide a possible mechanism of how transplanted ENSC remodel the ECM, to allow invasion of donor cells. Of note, early emigration of neural crest cells from the neural tube has been shown to be dependent on Mmp9^47^ and is associated with discontinuity of Collagen IV staining^37^. Similarly, here we demonstrate that major remodelling of the musculature and Collagen IV occurs, after ex vivo transplantation, around the site of neurosphere engraftment which appears to be consistent with neural crest emigration after transplantation. Yet, our findings of increased Collagen (*Col1a and Col4a*) gene expression were unexpected. Previous investigations have, however, demonstrated dynamic regulation of Collagen I and IV during neural crest cell migration. In particular, Collagen I expression has been shown to be upregulated along neural crest migratory pathways, whereas Collagen IV has been shown to be increased upon neural crest cell aggregation and differentiation^37^. Hence, these processes may account for the observations in the current study, as transplanted ENSC have been shown to migrate extensively within ex vivo transplanted tissue, and aggregate within endogenous ganglia structures at the level of the myenteric plexus.

While this study has revealed the timescale and a possible mechanism underlying donor cell integration within host tissues following ENSC transplantation, future studies will be required to fully elucidate the mechanisms involved. Here, we utilised early post-natal (P5-7) ENSC to investigate donor cell integration within C57BL/6J wild-type colon. A recent study has shown that enteric neural crest cells progressively lose capacity to form the ENS with age^32^. Hence, further studies will be required to determine the integration capacity of aged donor cells. This caveat may have significant implications in the clinical translation of cellular therapies for enteric neuropathies, as donor age will need to be considered for both autologous cell transplants, and in strategies using pluripotent cell sources; given time in culture may alter integrative capacity. Similarly, studies investigating alternative donor cell forms (i.e., neurospheres vs single cells) may be beneficial in determining the ideal cell “product” required for integration. Importantly, the outlined ex vivo transplantation methodology may offer significant benefits in tackling such questions in a medium-throughput, and consistent, manner. Furthermore, we used C57BL/6J wild-type colon as recipient tissue in this proof-of-principle study. We demonstrate that donor-derived cells infiltrate and “home” to the myenteric ganglia by three weeks post-transplantation. We assume that trophic factors regulate this “homing” process, though the exact factors involved remain unclear. Moreover, recent studies have demonstrated that the colonic ECM is altered in diseased states^48–50^, which suggests the integrative ability of ENSC-derived donor cells is likely to be altered by the recipient microenvironment in different disease states. Future studies will therefore be required to address how disease-specific parameters alter ENSC integration, which may be clinically relevant.

## Conclusions

We conclude that this study provides critical evidence on the timescale and mechanisms which regulate ENSC integration within recipient gut tissue. Furthermore, the modified organotypic culture model utilised in the current study may be beneficial in the future assessment of donor cell transplantation in neuropathic models, or in the development of targeted approaches to influence donor cell behaviour as a possible combinatorial strategy for the treatment of enteric neuropathies.

## Supplementary Information

Supplementary Legends.

Supplementary Information 1.

Supplementary Video 1.

Supplementary Video 2.

## Data Availability

All authors had access to the study data and had reviewed and approved the final manuscript. The data that support the findings of this study are available from the corresponding author upon reasonable request.

## Abbreviations

3D: Three-dimensional
ANOVA: One-way analysis of variance
CAM: Chick chorioallantoic membrane
Col1a: Collagen 1a2
Col4a: Collagen 4a1
D: Day (in culture)
DMEM: Dulbecco’s modified eagle medium
ECM: Extracellular matrix
EGF: Epidermal growth factor
Eln: Elastin
EMT: Epithelial-mesenchymal transition
ENS: Enteric nervous system
ENSC: Enteric neural stem cells
FACS: Fluorescence activated cell sorting
FGF: Fibroblast growth factor
Fn1: Fibronectin 1
FPS: Frames per second
Gapdh: Glyceraldehyde 3-phosphate dehydrogenase
GFAP: Glial fibrillary acidic protein
GFP: Green fluorescent protein
Lama1: Laminin a1
Lamb1: Laminin b1
Mmp1: Matrix metalloproteinase 1
Mmp2: Matrix metalloproteinase 2
Mmp8: Matrix metalloproteinase 8
Mmp 9: Matrix metalloproteinase 9
Mmp13: Matrix metalloproteinase 13
NSM: Neurosphere medium
ODU: Optical density units
PBS: Phosphate-buffered saline
qRT-PCR: Quantitative real-time polymerase chain reaction
ROI: Region of interest
RT: Room temperature
RT-PCR: Real-time polymerase chain reaction
SMA: Smooth muscle actin
SM22: Smooth muscle protein 22 alpha
TuJ1: Neuron-specific class III beta-tubulin
YFP: Yellow fluorescent protein
