# Supplementary material for: Dynamic integration of enteric neural stem cells in ex vivo organotypic colon cultures
Source: Sci Rep. 2021 Aug 5;11:15889. doi: 10.1038/s41598-021-95434-4 (PMC8342505; doi:10.1038/s41598-021-95434-4)
Supplement: Supplementary file 1 — Supplementary Legends. [file 41598_2021_95434_MOESM1_ESM.docx]

**Supplementary Figure 1. Non-uniform migration of ENSC in recipient colon following *ex vivo* transplantation. (A)** Representative low-power brightfield image, taken at transplantation, showing mounted C57BL/6J colonic tissue with transplanted neurosphere *in situ* (*arrowhead*). **(B)** Representative z-stacked image showing the migration of donor YFP^+^ ENSC-derived cells (arrows) away from the site of neurosphere integration 1-day after *ex vivo* transplantation. Scale bar represents 100μm.

**Supplementary Figure 2. Early integration and migration of ENSC in recipient colon following *ex vivo* transplantation. (A-C)** Representative fluorescent images showing the development of YFP^+^ donor cells within 3 independent recipient C57BL/6J colonic tissue 3-days after *ex vivo* transplantation. Note that migration away from the transplanted neurosphere occurs in all directions as indicated by *white arrows*. Scale bar represents 50μm.

**Supplementary Figure 3. Migration of transplanted ENSC across the *muscularis*. (A)** Partial confocal z-stack image of GFP^+^ (*green*) ENSC-derived cells expressing HuC/D (*red*) at the serosal aspect 21-days after *ex vivo* transplantation. **(B)** Partial confocal z-stack of myenteric plexus region, lying immediately below that shown in A in *z,* showing GFP^+^ (*green*) ENSC-derived cell bodies (*blue; DAPI*) have migrated across the *muscularis* to integrate within, or lie in close association with, the endogenous myenteric ganglia which express HuC/D (*red*). See Supplementary Movie 1 for 3D reconstruction of entire confocal z-stack. Scale bar represents 50μm.

**Supplementary Figure 4. Assessment of ENSC proliferation *in vitro* and after *ex vivo* transplantation. (A)** Representative fluorescent image demonstrating the presence of the proliferative marker Ki67 (*red*) within *Wnt1^cre/+^;R26R^YFP/YFP^*-derived neurospheres; GFP (*green*); DAPI (*blue*). Note the presence of multiple Ki67^+^ cells within neurospheres *in vitro*. **(B&C)** Representative confocal z-stack images of GFP^+^ (*green*) ENSC-derived cell bodies (DAPI; *blue*), within the gut wall 3-days (*B*) and 21-days (*C*) after *ex vivo* transplantation. Note the apparent absence of co-expression of Ki67 in GFP^+^ cells at both early and late timepoints. Scale bars represent 50μm.

**Supplementary Figure 5. Assessment of ENSC apoptosis *in vitro* and after *ex vivo* transplantation. (A)** Representative fluorescent image demonstrating the presence of the apoptotic marker cleaved Caspase 3 (*red*) within *Wnt1^cre/+^;R26R^YFP/YFP^*-derived neurospheres; GFP (*green*); DAPI (*blue*). Note the presence of multiple Caspase 3^+^ cells within neurospheres *in vitro*. **(B&C)** Representative confocal z-stack images of GFP^+^ (*green*) ENSC-derived cell bodies (DAPI; *blue*), within the gut wall 3-days (*B*) and 21-days (*C*) after *ex vivo* transplantation. Note the apparent absence of co-expression of Caspase 3 in GFP^+^ cells at both early and late timepoints. Scale bars represent 50μm.

**Supplementary Figure 6.** **Disruption of smooth muscle actin expression following *ex vivo* transplantation. (A)** Representative merged confocal z-stack images showing GFP^+^ (*green*) ENSC-derived cells and endogenous SMA^+^ (*red*) smooth muscle within C57BL/6J colonic tissue 21-days following *ex vivo* transplantation. (**B&C**) Individual channels, from *A,* showing GFP^+^ (*green; B*) ENSC-derived cells and endogenous SMA^+^ (*red, C*) smooth muscle. Note the disruption of SMA expression at the site of neurosphere engraftment (*magenta border*; *C*).

**Supplementary Figure 7. Matrix metalloproteinase gene expression in *Wnt1^cre/+^;R26R^YFP/YFP^*-derived neurospheres.** Representative RT-PCR gel demonstrating expression of candidate *Mmp* genes within YFP^+^ neurospheres. Note the presence of *Mmp2* and *Mmp9* compared to the absence of *Mmp8* and *Mmp13. Gapdh:* house-keeping gene.

**Supplementary Movie 1. Transplanted ENSC integrate across the colonic *muscularis* following *ex vivo* transplantation.** Representative video (*15 fps*) of 3D-reconstructed confocal micrograph showing GFP^+^ (*green*), HuC/D_­_^+^ (*red*) and DAPI^+^ (*blue*) to show integration of donor GFP^+^ cells across the outer gut wall and association with HuC/D^+^ endogenous neurons at the level of the myenteric ganglia. See Supplementary Figure 3 for confocal z-stacked images of serosal and myenteric regions.

**Supplementary Movie 2. Transplanted ENSC integrate extensively across the colonic wall following *ex vivo* transplantation.** Video (*15 fps*) of depth-coded confocal micrograph demonstrating integration of ENSC-derived GFP^+^ donor cells across the gut wall, in C57BL/6J colonic tissue, 21-days after *ex* vivo transplantation. Initially, GFP^+^ donor cells can be seen to have migrated extensively, in both the longitudinal and circumferential directions, from the engraftment site. Depth-coding of this GFP^+^ network shows the integration of ENSC-derived GFP^+^ cells at variable depths within the *tunica muscularis.*
