# Supplementary material for: Dynamic integration of enteric neural stem cells in ex vivo organotypic colon cultures
Source: Sci Rep. 2021 Aug 5;11:15889. doi: 10.1038/s41598-021-95434-4 (PMC8342505; doi:10.1038/s41598-021-95434-4)
Supplement: Supplementary file 2 — Supplementary Information 1. [file 41598_2021_95434_MOESM2_ESM.docx]

DYNAMIC INTEGRATION OF ENTERIC NEURAL STEM CELLS IN EX VIVO ORGANOTYPIC COLON CULTURES.

Georgina Navoly and Conor J. McCann*

**Supplementary Figure. 1**


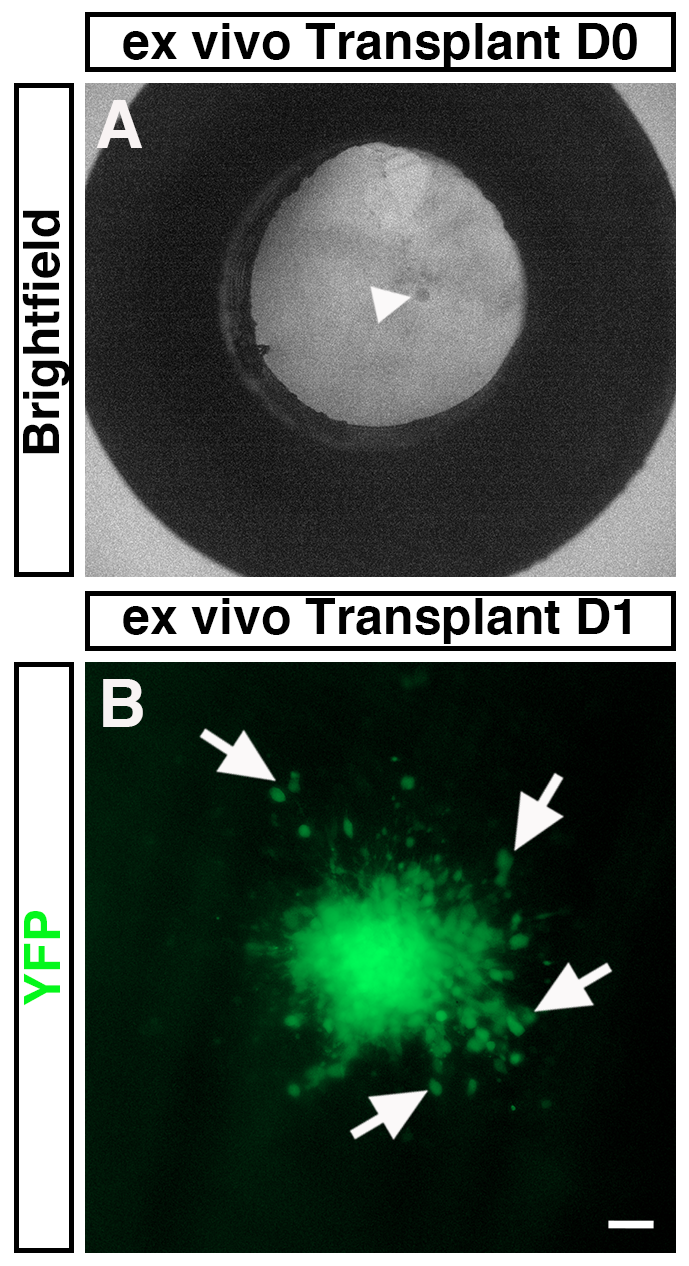


**Supplementary Figure. 2**

**
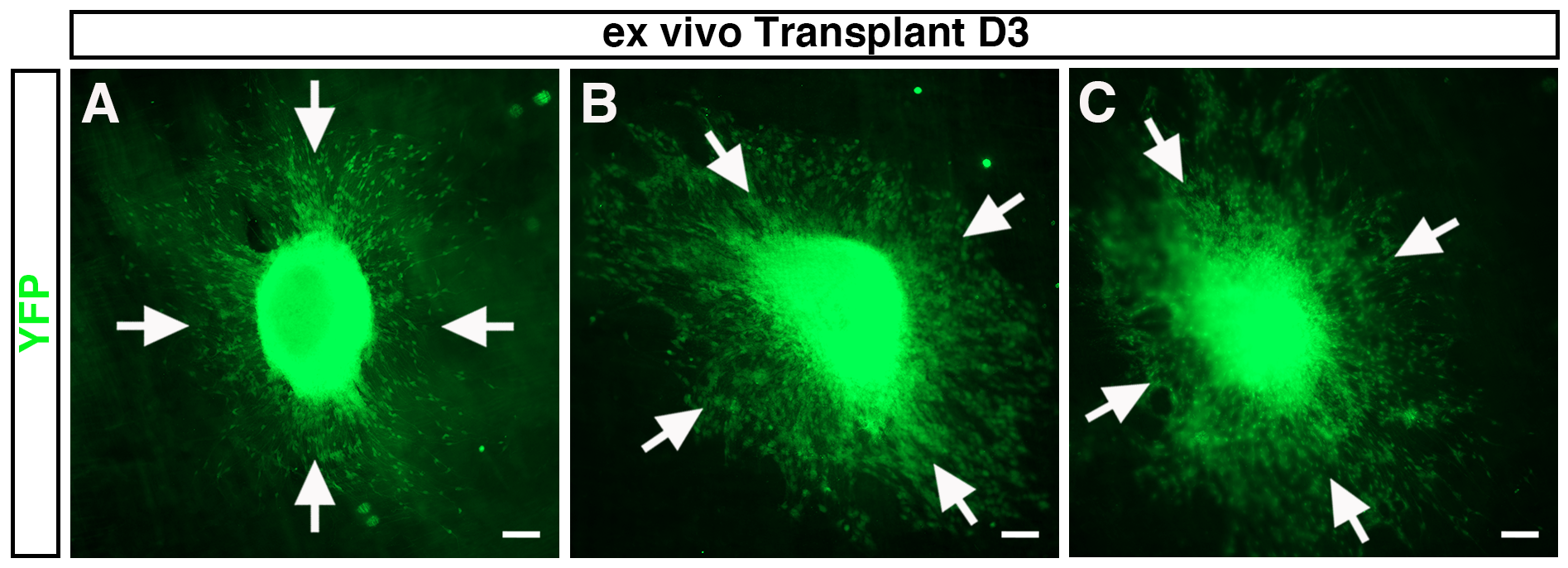
**

**Supplementary Figure. 3**

**
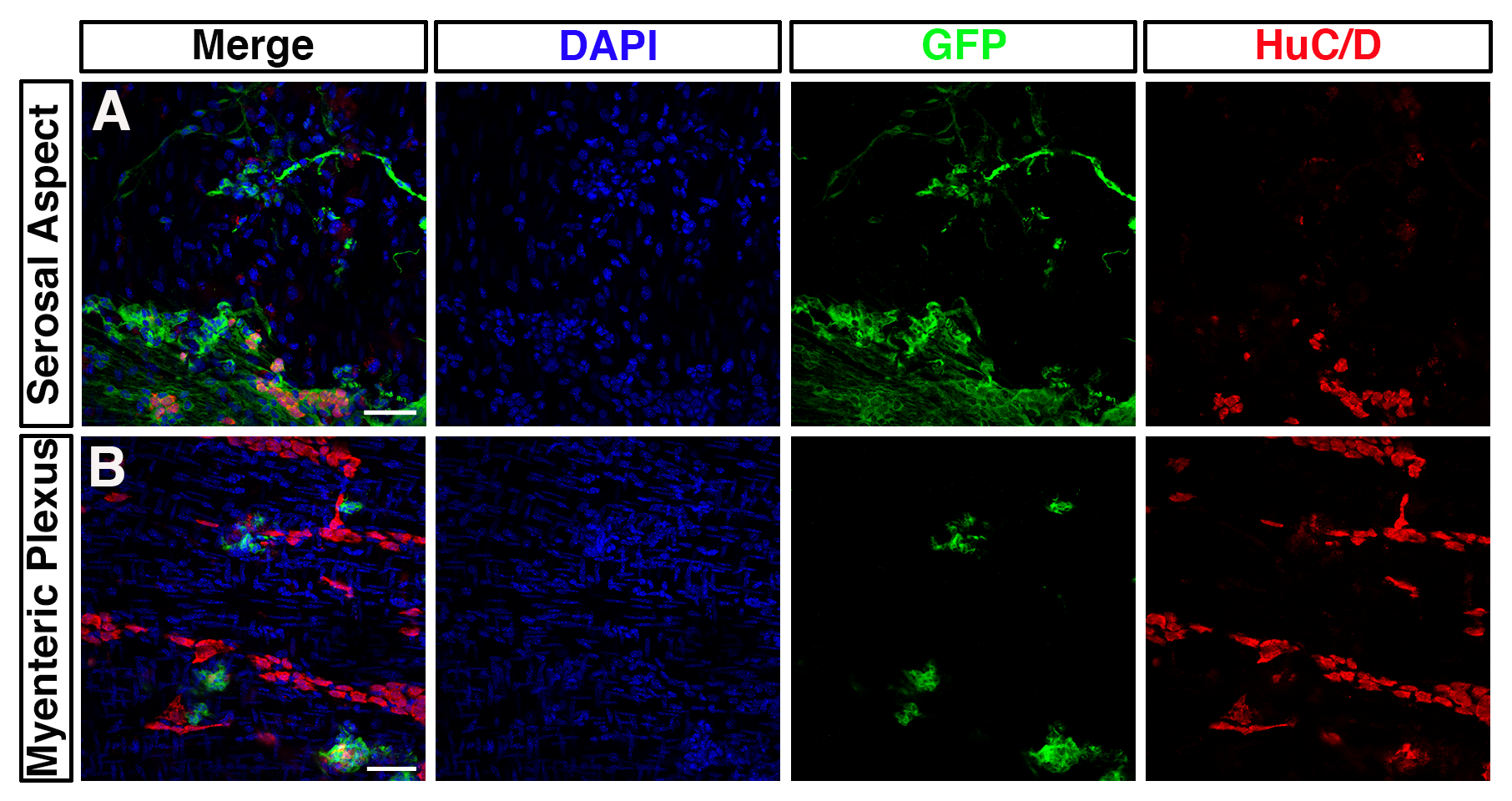
**

**Supplementary Figure. 4**

**
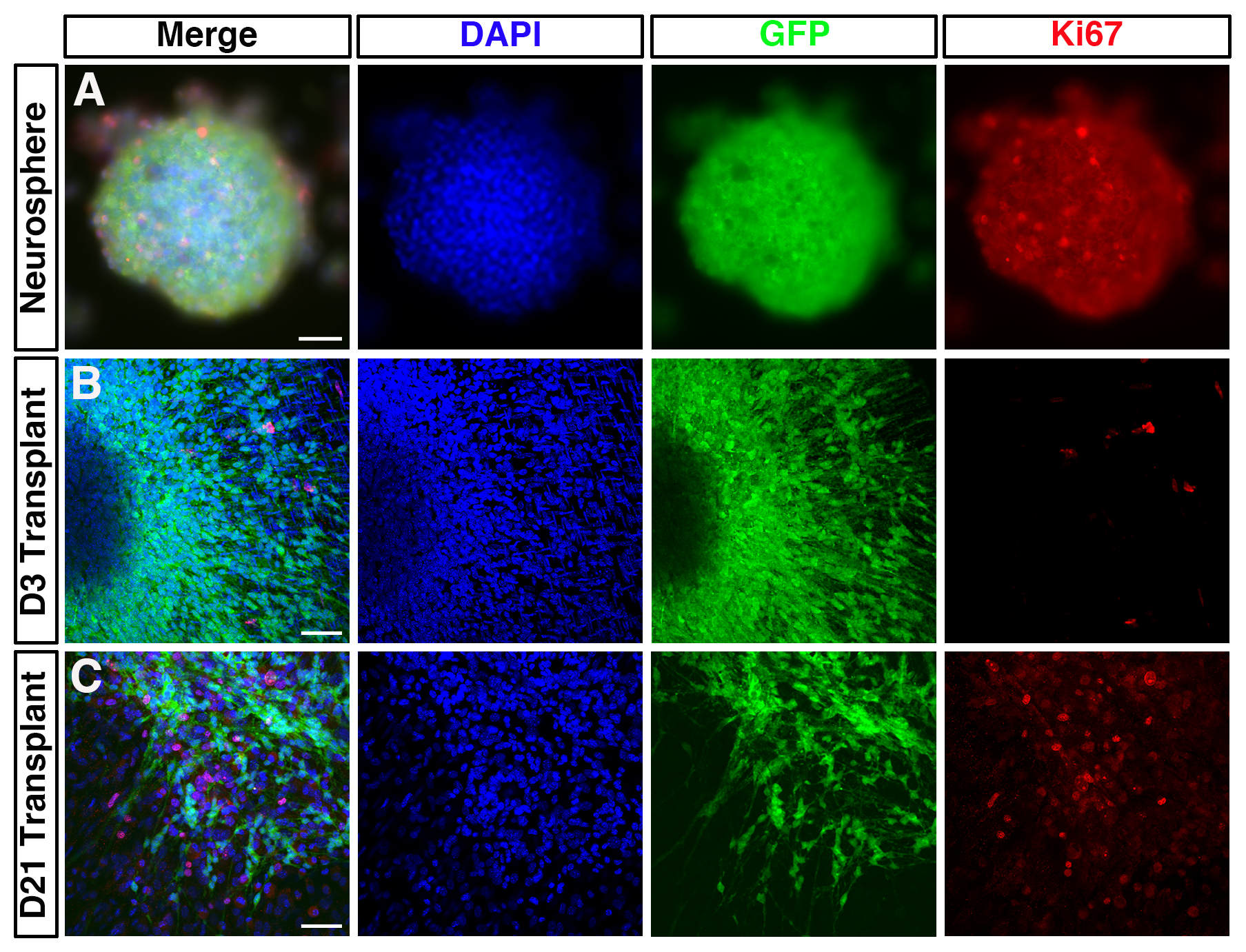
**

**Supplementary Figure. 5**

**
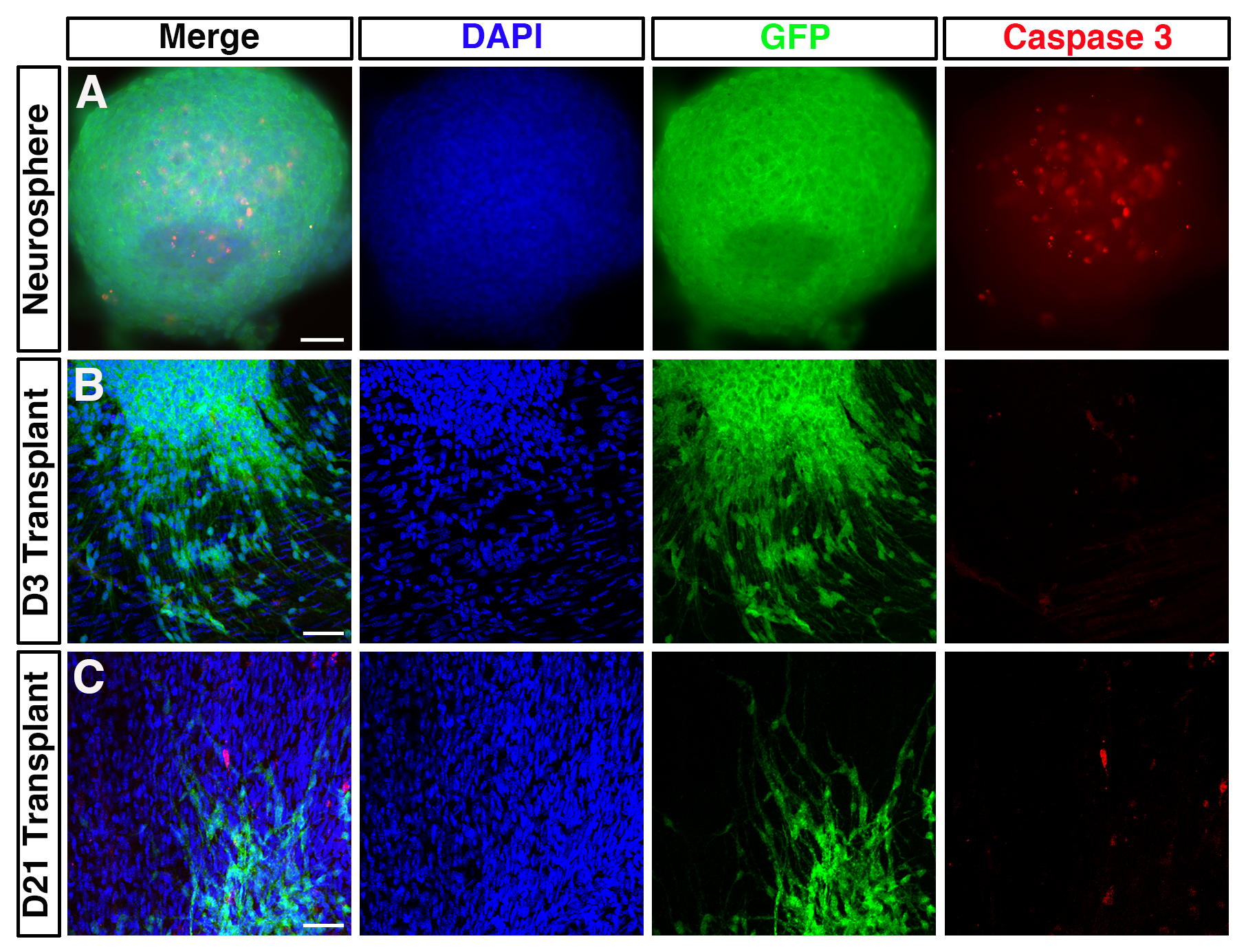
**

**Supplementary Figure. 6**

**
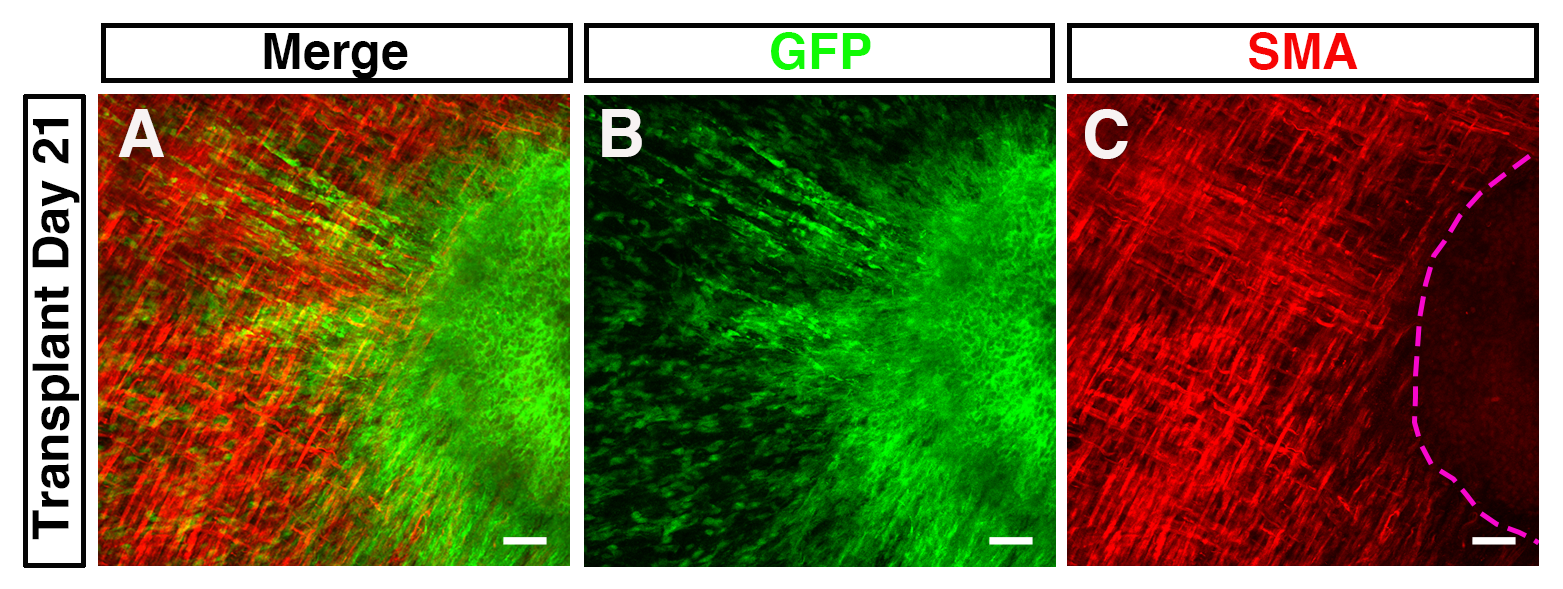
Supplementary Figure. 7**

**
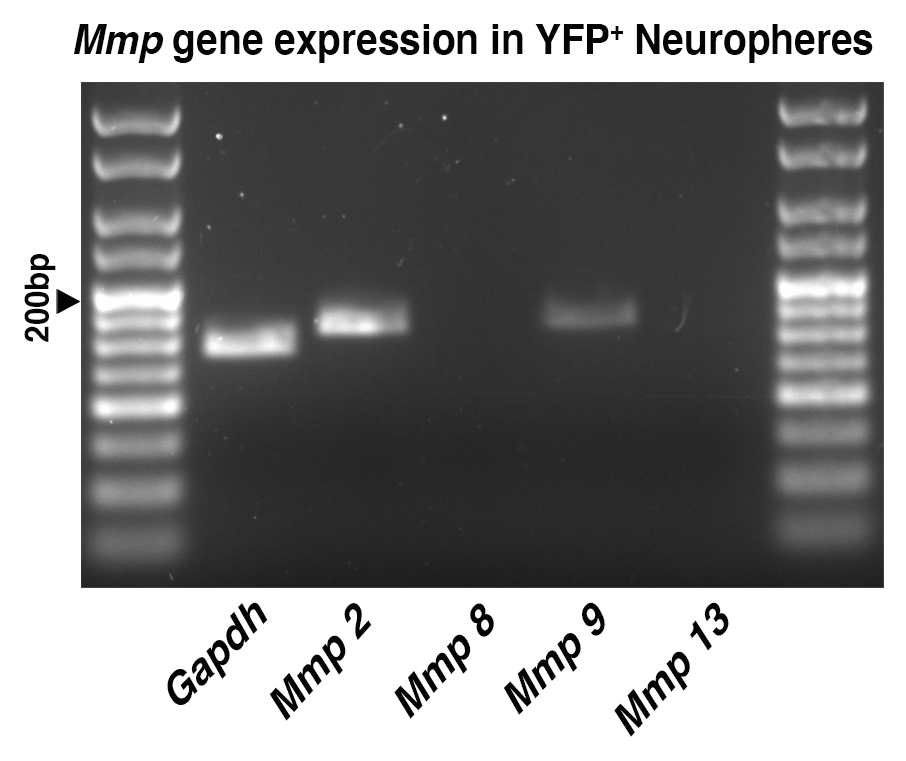
**

**Supplementary Table 1. Primary Antibodies used for Immunohistochemistry**

| **Primary Antibody** | **Concentration** | **Company** |
| --- | --- | --- |
| Mouse anti-TuJ1 | 1:500 | Covance |
| Rabbit anti-GFAP | 1:500 | Sigma-Aldrich |
| Mouse anti-GFP | 1:500 | Invitrogen |
| Rabbit anti-GFP | 1:500 | Invitrogen |
| Rabbit anti-SM22 | 1:500 | Abcam |
| Rabbit anti-SMA | 1:500 | Sigma-Aldrich |
| Rabbit anti-HuC/D | 1:500 | Invitrogen |
| Rabbit anti-Ki-67 | 1:500 | Novocastra |
| Rabbit anti- cleaved Caspase 3 | 1:1000 | Cell Signalling Technologies |
| Rabbit anti-Collagen IV | 1:200 | Abcam |

**Supplementary Table 2. Secondary Antibodies used for Immunohistochemistry**

| **Secondary Antibody** | **Alexa Fluor** | **Concentration** | **Company** |
| --- | --- | --- | --- |
| Goat anti-mouse | 488 | 1:500 | Invitrogen |
| Goat anti-mouse | 568 | 1:500 | Invitrogen |
| Goat anti-rabbit | 488 | 1:500 | Invitrogen |
| Goat anti-rabbit | 568 | 1:500 | Invitrogen |
| DAPI | - | 1:1000 | Sigma |

**Supplementary Table 3. Primer Sequences used for RT-PCR**

| **Gene** | **Forward primer** | **Reverse primer** |
| --- | --- | --- |
| mCol1a2 | ACTAAGTTGGAGGGAACGGT | AGAGTCCGCGTATCCACAAA |
| mCol4a1 | GTCTCTGCTGGTCCCCTG | CAGAGCCACCACAATCGC |
| mFn | AAATCGTGCAGCCTCAATCC | GTTAAAACCCCGGCTTCCTC |
| mLama1 | TTCCCTGCCATTCTCAACCT | TGTTACCGTCACAGACCCG |
| mLamb1 | TTTTCTCCCCGCTACCTCTC | TAGGACACCAAAGGCGAACA |
| mEln | CCTACCAGGCAGCAATTACG | TGGAGGAGGTTGAGCAAGAG |
| mMmp1 | GAGACCGGCAAAATGTGGAG | TCCAGTCACTTTCAGCCCAA |
| mMmp2 | CCCCGATGCTGATACTGACA | GGTGTCACTGTCCGCCAAAT |
| mMMP8 | CCAATGCCTTCCCAGTACCT | GCGCTGCATCTCTTTAAGCT |
| mMmp9 | TAGATCATTCCAGCGTGCCG | TGTGGTTCAGTTGTGGTGGT |
| mMmp13 | CCCCTTCCCTATGGTGATGA | TTTCTCGGAGCCTGTCAACT |
| Gapdh | TCCTGCACCACCAACTGCTT | CACGCCACAGCTTTCCAGAG |
